# Community-based mortality surveillance among internally displaced vulnerable populations in Banadir region, Somalia, 2022–2023

**DOI:** 10.3389/fpubh.2025.1582558

**Published:** 2025-04-09

**Authors:** Mohamed Hussein Adam, Bashiru Garba, Hassan Abdullahi Dahie, Joaquin Baruch, Jonathan A. Polonsky, Jihaan Hassan, Jamal Hassan Mohamoud, Dahir Abdi Ali, S. K. Md Mamunur Rahman Malik, Francesco Checchi, Najib Isse Dirie

**Affiliations:** 1 Department of Public Health, Faculty of Medicine and Health Sciences, SIMAD University, Mogadishu, Somalia; 2 SIMAD Institute for Global Health (SIGHt), SIMAD University, Mogadishu, Somalia; 3 Department of Veterinary Public Health and Preventive Medicine, Faculty of Veterinary Medicine, Usmanu Danfodiyo University, Sokoto, Nigeria; 4 Nursing and Midwifery Department, Faculty of Medicine and Health Sciences, SIMAD University, Mogadishu, Somalia; 5 World Health Organization, Regional Office for the Eastern Mediterranean, Cairo, Egypt; 6 Geneva Centre of Humanitarian Studies, Faculty of Medicine, University of Geneva, Geneva, Switzerland; 7 Department of Paediatrics and Child Health, Dr. Sumait Hospital, Faculty of Medicine and Health Sciences, SIMAD University, Mogadishu, Somalia; 8 Faculty of Economics, SIMAD University, Mogadishu, Somalia; 9 Faculty of Epidemiology and Population Health, London School of Hygiene and Tropical Medicine, London, United Kingdom; 10 Department of Urology, Dr. Sumait Hospital, Faculty of Medicine and Health Sciences, SIMAD University, Mogadishu, Somalia

**Keywords:** Somalia, humanitarian crisis, internally displaced persons, community-based surveillance, verbal autopsy, mortality rates, malnutrition, infectious diseases

## Abstract

Somalia faces a severe humanitarian crisis driven by conflict, drought, and rising food prices, straining its fragile health system. Internally displaced persons (IDPs) suffer high mortality rates, yet data on causes of death remain limited. This study integrates verbal autopsy (VA) with community-based surveillance (CBS) to identify mortality causes in IDP populations. A hybrid retrospective-prospective mortality surveillance study was conducted in 57 IDP camps across Daynile and Kahda districts, Banadir region, from October 2022 to November 2023. Retrospective baseline data from 20,323 individuals were collected in January–February 2023, followed by prospective surveillance rounds in March, April, and May–November 2023. Causes of death were determined using WHO-standardized VA methods. During the retrospective period, Daynile had a CDR of 3.15 per 10,000 person-time, while Kahda’s was 1.26. Mortality rates fluctuated, showing significant reductions at certain times. Over the prospective data collection period, the overall CDR was 0.64 per 10,000 person-time. Verbal autopsies revealed that severe malnutrition, respiratory infections, and diarrheal diseases were the leading causes of death. Among children under five, malnutrition, measles, and neonatal pneumonia were the primary causes. Our study highlights the severe impact of malnutrition and infectious diseases on mortality rates among IDPs in Banadir. Continuous surveillance and targeted health interventions are crucial to address the ongoing humanitarian crisis in Somalia. Enhancing training for data collectors and fostering community engagement can improve data accuracy and support timely humanitarian responses.

## Introduction

Somalia is gripped by a prolonged humanitarian crisis fueled by conflict, recurrent droughts, economic fragility, and the COVID-19 pandemic. The 2011–2012 famine claimed 258,000 lives, half of whom were children under five (1). Despite intermittent interventions, acute food insecurity and displacement persist, exacerbated by deteriorating security and restricted humanitarian access (2). In late 2021, four consecutive seasons of below-average rainfall triggered a renewed food insecurity crisis. The crisis resulted in an estimated 43,000 excess deaths during 2022, with more projected to die during 2023 (3). In addition to pervasive food insecurity, Somalia’s healthcare system remains critically impaired by inadequate WASH infrastructure, insufficient immunization coverage, and fragile disease surveillance (4). Mortality statistics primarily rely on health facility records, which capture only a fraction of deaths, as most occur outside formal medical settings (2). Although the Integrated Disease Surveillance and Response (IDSR) system was introduced to enhance mortality tracking, its implementation is hindered by resource limitations and weak integration with community-based reporting (5), resulting in significant data gaps that impede accurate mortality assessments (4). While district-level retrospective Standardized Monitoring and Assessment of Relief and Transitions (SMART) surveys provide periodic mortality estimates, their infrequency and lack of specificity regarding causes of death limit their utility for humanitarian decision-making (6).

To address these surveillance gaps, Community-Based Surveillance (CBS) has emerged as a crucial approach for tracking mortality in crisis settings, utilizing trained local networks for real-time data collection and early detection of public health threats (7–9). CBS can enhance humanitarian response by ensuring timely interventions and improving resource allocation (5). Beyond its surveillance role, CBS may foster community engagement, empowering local populations to take an active role in health monitoring (7). Measurement of cause-specific mortality can be achieved by combining CBS with verbal autopsy, a questionnaire-based method to determine the probable cause of death in areas with limited access to advanced biomedical diagnostics (10). In this study, we combined CBS approaches with verbal autopsies with the objective of understanding the population dynamics (arrivals, exits, deaths, births) and causes of mortality among IDPs in the Banadir region of Somalia during the recent drought.

## Methods

### Study setting, population and period

A CBS was established in early 2023, coinciding with peak drought conditions. The study was done in internally displaced people’s (IDP) camps located in Banadir region which comprises the capital city Mogadishu (Supplementary Figures 1a–c). This region is home to the largest concentration of IDPs in Somalia with 1,979 verified IDP camps hosting 230,473 households and 1,247,669 individuals (11). The CBS was implemented in 57 camps, most of whose residents were displaced due to the latest drought, conflict and/or post-drought floods. The camps are in Daynile and Kahda districts (administrative level 2) of Banadir region (Mogadishu), selected as they host the largest concentration of IDPs. All households living in the camps at the time of data collection were considered eligible. Public health services in these camps were scarce, primarily provided by NGOs with minimal government involvement. In early 2023, WHO, in collaboration with the Ministry of Health, deployed integrated outreach teams to offer basic healthcare, routine immunization, and maternal and child health services (12). The start of the period over which data were collected was 1^st^ October 2022, and data were collected until November 2023.

### Data collection

During January–February 2023, baseline retrospective data was collected during an exhaustive survey. Aided by camp leaders, data collectors approached each head of household, explained the purpose of the surveillance, sought informed verbal consent, and administered a structured questionnaire; answers were entered onto electronic tablets using a Kobo-toolbox platform. The questionnaire collected demographic data on each individual present in the household during the period from 1 October 2022 to February 2023, including name, age in years, sex, whether the individual arrived at the camp before, on or after 1 October 2022, whether the individual joined or was born into the household during the recall period, or whether the individual left the household/died during the period. For individuals who died, the location of death (pre-displacement; during the displacement journey; in the camp) and cause of death (disease, injury, others) according to the next-of-kin was recorded.

Following baseline data collection, we collected data for three periods: (1) February–March 2023, (2) April 2023 and (3) May–November 2023. During periods 1 and 2, collectors contacted each camp’s designated leader every 10 days and asked whether any households in the camp had experienced new demographic events (joining, leaving, dead or newborn members) or whether an entire household had newly settled in the camp. If any such event was reported, the team of data collectors visited the camp and collected information on these events. Due to resource constraints this intensive data collection schedule was discontinued over the third period: instead, data collectors returned to the camps in October–November and collected information on households and individuals who had experienced any of the aforementioned dynamics.

Data collectors underwent training on survey administration, verbal autopsy procedures, ethical considerations, and electronic data entry. Validation measures included pre-testing the questionnaire, conducting pilot interviews, and supervisors implementing real-time spot checks, reviewing inconsistencies, and providing feedback to field teams. Additionally, automated validation in KoboToolbox flagged missing or inconsistent responses for real-time correction. Camp leaders and neighboring households helped verify demographic changes.

### Death rate estimation

The crude death rate (CDR), representing deaths from all causes among all age groups, and the under-five death rate (U5DR), specific to children under five, were calculated per 10,000 person-days over the entire study period. While the exact dates of events were recorded for rounds 2 and 3, they were not in the baseline and round 1. Therefore, we computed person-time at risk for the baseline and round 1 sub-periods by assuming that events occurred at the midpoint of the recall period.

### Verbal autopsy data collection and analysis

Following baseline data collection, field teams revisited households that had reported a death to administer the standardized World Health Organization (WHO) verbal autopsy (VA) questionnaire (13). The questionnaire was administered by two medical doctors.

To determine probable causes of death, VA data were analyzed using the InsilicoVA algorithm within the openVA R package (14). This Bayesian probabilistic model infers mortality causes based on symptom patterns, offering a standardized, scalable alternative to physician-certified VA. A total of 10,000 simulations were run, both overall and stratified by age group (<5 years and ≥ 5 years): we present the mean and 95% percentile interval of simulations.

## Results

### Description of the population

During baseline data collection, 20,323 people were registered, with 9,133 (44.9%) in Daynile and 11,190 (55.1%) in Kahda (Table 1). Across both districts, 21.3% of household members were aged 0–4 years (Daynile: 21.7%; Kahda: 21.0%). Age-sex distributions by demographic status are shown in Supplementary Figure S2. Deaths were disproportionately concentrated among children under five and adults over 60. Most instances of migrating in or out of households were among younger or middle-aged adults, plausibly reflecting mobility trends driven by conflict, food insecurity, and economic pressures. In contrast, those who remained (‘Remained’) exhibited a broader age distribution. These demographic patterns remained consistent across surveillance rounds.

**Table 1:** Number (%) of HH members ever-present during the recall period (baseline) by age and district.

Age group	Daynile	Kahda	Total
0–4	21.7% (1,981)	21.0% (2,352)	21.3% (4,333)
5+	78.3% (7,152)	79.0% (8,838)	78.7% (15,990)
Total	9,133 (44.9%)	11,190 (55.1%)	20,323 (100%)

A total of 722 deaths were recorded. The majority (64.9%) occurred at the current location, with a further 17.2% during migration, while 16.7% occurred in the pre-displacement residence (Supplementary Table S1).

### Mortality estimates

During the baseline period, Daynile exhibited a higher CDR than Kahda (3.15 vs. 1.26); CDR declined in both districts during period 1 but spiked again in Kahda during period 2, with the mean CDR remaining around 0.6 in both districts in period 3. Corresponding estimates for U5DR are also shown in Table 2, suggesting exceptionally high child mortality in Kahda and especially Daynile during the October 2022–February 2023 period.

**Table 2:** Estimated crude death rates (CDR) and under 5 years death rates (U5DR) per 10,000 person-days, by period.

District	All-age deaths			Deaths among children under five		
	Number of deaths	Person-time (days)	CDR	Number of deaths	Person-time (child days)	U5DR
Oct 2022 to Jan–Feb 2023						
Daynile	340	1,080,636	3.15	162	229,782	7.05
Kahda	103	817,112	1.26	46	170,155	2.70
Total	443	1,897,749	2.33	208	399,938	5.20
March 2023						
Daynile	12	258,848	0.46	5	57,808	0.86
Kahda	12	351,888	0.34	7	74,768	0.94
Total	24	610,736	0.39	12	132,576	0.91
April 2023						
Daynile	14	246,167	0.60	0	51,064	0.00
Kahda	39	312,563	1.25	1	67,027	0.15
Total	53	558,730	0.90	1	118,091	0.08
May–Nov 2023						
Daynile	86	1,377,438	0.62	36	307,272	1.17
Kahda	116	1,791,951	0.65	47	385,197	1.22
Total	202	3,169,389	0.64	83	692,469	1.20

**Figure 1:**
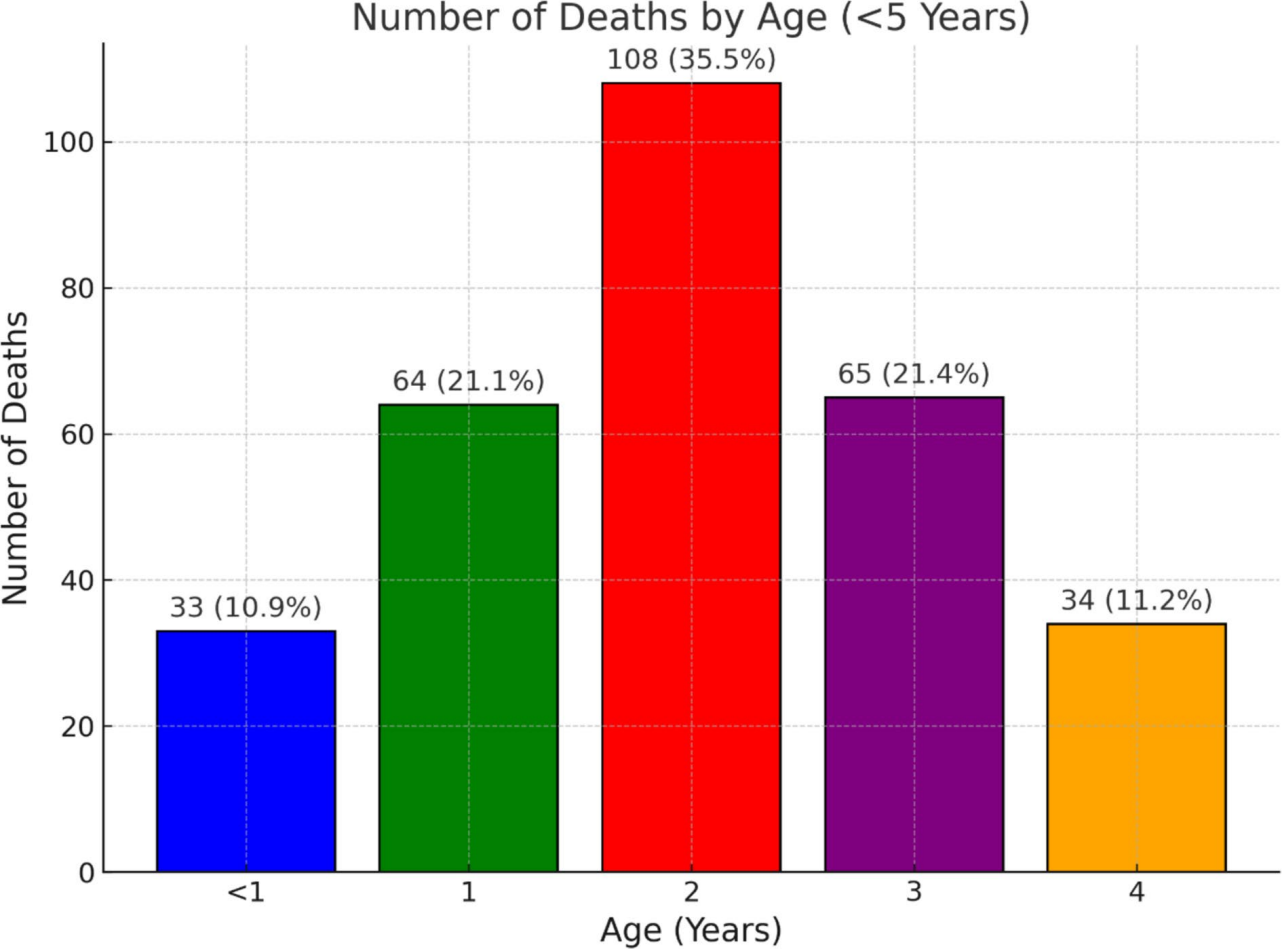
Age distribution of deaths among children under five across all data collection rounds.

Figure 1 illustrates the age distribution of deaths among children under five, with the highest proportion of deaths at age 2 (35.5%), followed by ages 1 (21.1%) and 3 (21.4%). Mortality is lower among infants under 1 year (10.9%) and declines further by age 4 (11.2%).

### Verbal autopsy

**Figure 2:**
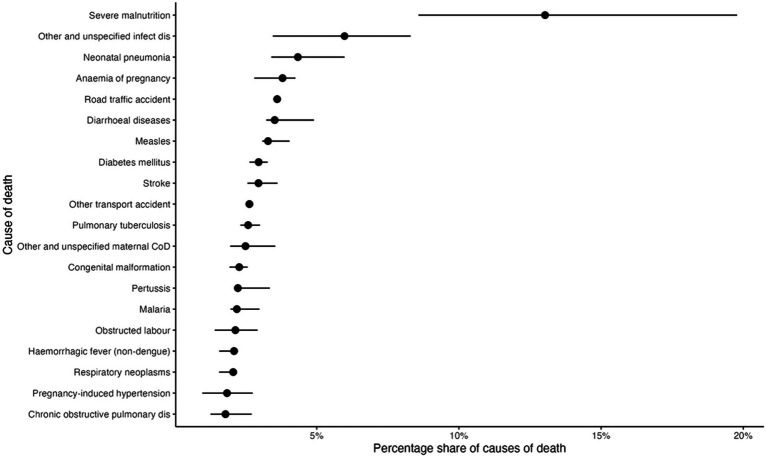
Estimated percentage share of causes of death (*n* = 722). Black dots indicate the point estimate, while horizontal segments denote the 95% percentile interval.

Out of the 722 recorded deaths, severe malnutrition emerged as the leading cause of mortality, followed by unspecified infectious diseases, neonatal pneumonia, anemia of pregnancy, and road traffic accidents (Figure 2).

**Figure 3:**
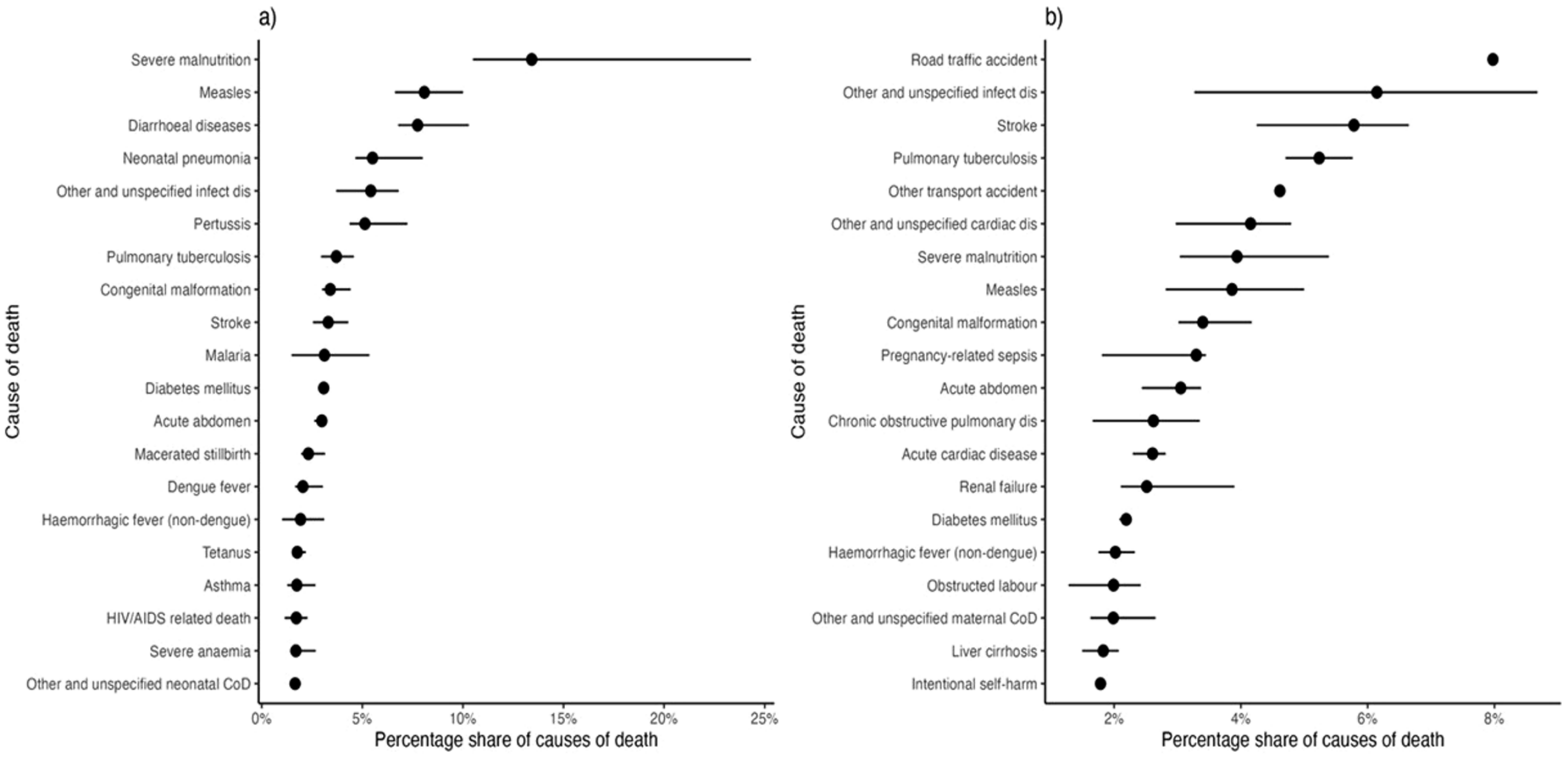
Estimated percentage share of causes of death among children under five (panel **a**) and older persons (panel **b**). Black dots indicate the point estimate, while horizontal segments denote the 95% percentile interval.

Among children under five, severe malnutrition, measles, diarrheal diseases, neonatal pneumonia, and unspecified infectious diseases are the predominant causes of death (Figure 3a). In older persons (18 years and above), road traffic accidents, unspecified infections, stroke, pulmonary tuberculosis and other transport injuries account for the highest mortality burden (Figure 3b).

Neonatal (up to 28 days of life) deaths were mostly attributable to neonatal pneumonia, measles, malnutrition and diarrhoeal diseases (Supplementary Figure S3), while over 5 years, tuberculosis, road traffic injuries, and stroke are leading causes (Supplementary Figure S4).

## Discussion

This study provides critical insights into mortality patterns among internally displaced populations (IDPs) in Somalia, highlighting the complex interplay between food insecurity, infectious diseases, trauma-related deaths, and healthcare system deficiencies. The findings from the present study, which assessed Crude Death Rates (CDR) and Under-5 Death Rates (U5DR) in Daynile and Kahda districts over a 1-year period, reveal significant disparities in mortality, particularly among children under five. From October 2022 to February 2023, Daynile experienced notably higher CDR (3.15 deaths per 10,000 person-time) and U5DR (7.05 deaths per 10,000 person-time), indicating a severe mortality burden compared to Kahda, which had a CDR of 1.26 deaths per 10,000 person-time and a U5DR of 2.70 deaths per 10,000 person-time (Table 2). Several factors contribute to these differences, with Daynile facing greater challenges due to its role as a primary settlement area for displaced persons, particularly from Lower Shabelle. These displaced populations often arrive in critical condition, and the resource strain from the large number of IDP camps further exacerbates food insecurity and healthcare shortages, increasing mortality rates. These rates were much higher than those found in Somalia during relatively favorable periods between droughts, where the countrywide CDR was estimated to be between 0.33 and 0.38 deaths per 10,000 person-days, with the U5DR nearly twice as high (3).

By March 2023, both districts showed a marked reduction in mortality, with Daynile’s CDR falling to 0.46 deaths per 10,000 person-time and U5DR to 0.86 deaths per 10,000 person-time, and Kahda’s rates dropping to 0.34 deaths per 10,000 person-time and 0.94 deaths per 10,000 person-time, respectively. This reduction reflects the impact of the WHO, in collaboration with the Ministry of Health, deploying integrated outreach teams in early 2023 to provide basic healthcare, routine immunization, and maternal and child health services (12). These improvements align with findings from a separate study conducted in Ethiopia, where the CDR averaged 1.0 (95% CI: 0.8–1.2) deaths per 10,000 person-days over an analysis period, with site-specific CDRs ranging from 0.3 to 2.1 deaths per 10,000 person-days (15). The Ethiopian study observed the highest mortality rates during the rainy season (May to September) and in November, likely due to seasonal outbreaks of diseases like cholera, which commonly affect vulnerable populations in these periods. In comparison, the decreases in mortality observed in both Daynile and Kahda by March 2023 suggest a more favorable trend, although still elevated compared to non-crisis periods, as evidenced by the Ethiopian and Somali studies. Further improvements were noted in the subsequent months, with Daynile and Kahda both showing CDRs of c and 0.65, respectively, in the May–November 2023 period. However, child mortality remained a significant concern, with U5DRs of 1.17 in Daynile and 1.22 in Kahda. This persistent child mortality, despite an overall decline in CDR, reflects ongoing vulnerabilities, particularly among children, who remain highly susceptible during crises.

The shortage of children aged 10–15 years among those who remained is a striking deviation from the expected age distribution (Supplementary Figure S2). Typically, in a stable population, one would expect a pyramid-shaped structure with larger numbers of younger individuals. A possible explanation for this pattern is the long-term impact of the 2010–2012 famine (1), which led to high child mortality, particularly in areas that contribute to displacement into Mogadishu. This could have resulted in a “missing generation” effect, where fewer individuals from that cohort survived to be represented in the current population. Additionally, the concentration of migration among younger and middle-aged adults suggests that economic pressures, conflict, and food insecurity remain key drivers of mobility. If migration is primarily undertaken by working-age individuals, it may have consequences for household stability, particularly for children and the older adults who are left behind. Meanwhile, the high mortality among the youngest children and older adult individuals underscores their vulnerability in crisis conditions. The consistency of these trends across multiple surveillance rounds suggests that these patterns are not incidental but rather reflective of ongoing demographic shifts in displacement settings.

The findings of this study are consistent with the NMS 2022 Phase 2 report (6), both highlighting persistent mortality risks in displacement settings. Supplementary Table S1 shows that the majority of deaths (64.9%) occurred at the current location, mirroring the NMS report’s findings that, despite declining crude and under-five death rates, mortality within IDP settlements remains a concern. Additionally, 17.2% of deaths transpired during migration, emphasizing the dangers of displacement, while 16.7% occurred at the previous residence, suggesting that deteriorating conditions in areas of origin continue to force movement toward urban centers. The NMS report similarly notes increased population movement away from IDP sites, though with unclear resettlement pathways, indicating ongoing risks both during transit and after relocation. The consistency of these patterns across both studies reinforces the urgent need for improved healthcare accessibility within settlements and along migration routes to mitigate preventable deaths at all stages of displacement.

The age distribution of under-five mortality in (Figure 1) highlights the influence of data collection timing on observed trends, as reflected in the distribution of deaths by period in (Table 2). The lower-than-expected neonatal and infant deaths in rounds 1 and 2 likely reflect the challenge of detecting mortality dynamics over short observation periods when the population remains stable. In contrast, the baseline and final rounds, spanning longer durations, captured a more complete picture of mortality trends. This pattern aligns with studies in crisis-affected settings, such as Somalia’s Afgooye corridor, where extended surveillance over 2 years (March 2016–March 2018) was crucial in identifying mortality exceeding emergency thresholds (16). Additionally, malnutrition may play a significant role in the elevated deaths among children aged 1–3, as the cumulative effects of undernutrition become more pronounced over time.

The primary causes of under-five mortality—malnutrition, pneumonia, diarrheal diseases, and measles—underscore the devastating effects of food insecurity, inadequate WASH, missed immunizations, and poor healthcare access (Figure 3a). National models estimate that 50% of drought-related deaths in Somalia occur in children under five (17), mirroring global patterns where nearly half of under-five deaths in crisis settings result from preventable diseases such as pneumonia, diarrhea, and malaria (18). A 2022 UNICEF report highlights the persistently high child mortality rates in fragile states, emphasizing the need for community-based healthcare, improved nutrition, and expanded immunization coverage (19). Our findings reinforce these concerns, demonstrating that malnutrition, infectious diseases, and vaccine-preventable illnesses remain the primary drivers of child mortality in Somalia.

Neonatal mortality remains a significant global health challenge, with infections accounting for approximately one-third of the estimated 2.5 million annual neonatal deaths, and pneumonia playing a major role in this burden (20). In this study, neonatal deaths were primarily attributed to pneumonia, congenital abnormalities, and severe malnutrition, indicating critical gaps in perinatal care, maternal nutrition, and early-life infection management (Supplementary Figures S2, S3). Pneumonia poses a severe risk in crisis settings, accounting for 22 and 30% of all child deaths in post-war Liberia (21), and Somalia (22), with case fatality rates of 12 and 2.1% among hospitalized children, respectively. Similarly, a multi-country UNHCR study found that pneumonia contributed to 20% of under-five deaths in refugee camps, with higher rates in African than in Asian camps. In contrast, Mache Tsadik et al., Rai et al., and Kalter et al., who employed a similar methodological approach, identified birth asphyxia as the leading cause of neonatal mortality, highlighting intrapartum care failures such as delayed delivery, lack of skilled birth attendants, and insufficient neonatal resuscitation (23–25). These variations underscore the need for context-specific interventions, particularly in displaced communities such as those in Banadir Region, where high pneumonia and malnutrition rates demand improved postnatal infection control, respiratory support, and nutritional interventions.

Among older individuals, the high burden of tuberculosis-related deaths suggests persistent challenges in disease surveillance, case detection, and treatment access, particularly within overcrowded and unsanitary displacement settings. Studies conducted in South Sudan (26), Syria (27, 28), and Afghanistan (29) support this finding, demonstrating that TB control efforts in crisis settings are severely impacted by weak healthcare infrastructure, lack of trained personnel, inconsistent drug supply, and displacement-related overcrowding, which increases TB transmission. Furthermore, these studies highlight that inadequate healthcare access in conflict zones results in treatment interruptions, leading to higher rates of drug-resistant TB and poor treatment outcomes. Additionally, research from other humanitarian settings has shown that poor patient-provider communication, lack of trust in healthcare services, and weak disease surveillance systems further hinder TB control efforts, all of which are relevant to the Somali context. The prominence of road traffic injuries highlights gaps in transport safety regulations, emergency medical response, and trauma care. Evidence from Kenya (30), India (31), Brazil (32) and Nigeria (33), highlights weak road safety policies, poor traffic law enforcement, and delayed emergency response, exacerbating road traffic injuries, while the burden of stroke and cardiovascular disease signals an emerging crisis in hypertension and chronic disease management, challenges intensify in crisis settings where healthcare disruptions, medication shortages, and weak infrastructure limit effective treatment. Studies highlight that conflict and displacement worsen hypertension control, increasing cardiovascular risks, while integrating chronic disease care into humanitarian response and ensuring medication access are crucial for reducing preventable deaths (34–36). Surprisingly, no violence-related deaths were reported despite the ongoing conflict. This may reflect the structured security within IDP camps, minimizing direct violence post-displacement. However, verbal autopsy (VA) limitations, including underreporting due to fear or stigma, may have influenced this finding.

Verbal autopsy (VA) is a crucial tool for determining causes of death in crisis settings where medical certification is unavailable (37). Several studies have attempted to assess the validity of VA tools (38–43). Nevertheless, its validity remains a challenge compared to full diagnostic autopsies and other methods. Notably, the emotional and cultural barriers often complicate interviews, making it essential to provide interviewers with specialized training in counseling and cultural sensitivity (44). The recall period varies widely, with delayed interviews risking memory loss and early ones causing distress, affecting participation and accuracy (45–47). The educational background of VA interviewers also influences the accuracy of data collection with some sites employing medical professionals and others relying on trained laypeople, potentially introducing bias (45, 46, 48). Analytical challenges persist, as physician review lacks repeatability and is time-consuming, while algorithm-based methods, though promising, remain inconsistent (42, 45, 49). Despite these challenges, VA continues to be a vital tool in global health, particularly important in economically constrained regions where full autopsies are unfeasible, relying on anamnestic data from relatives. Hart et al. (50) find it effective, with promising validation results. The effort of the scientific community has mainly focused on validating the technique by relying on medical records and reading/interpreting the data itself, which can be performed by physicians or through algorithms (51).

### Limitations

Underreporting of child deaths, particularly neonatal and infant, may have affected our estimates, particularly in periods 2 and 3 due to the reliance on camp leaders to detect these deaths and potential data collection fatigue. Thereafter, efforts were put in place to motivate the data collectors including recruitment of additional data collectors. Inconsistencies were noted in the reported locations of death among those who arrived in camps before October 2022: 124 of these deaths were reported as occurring during migration, 121 in the pre-displacement residence and 9 in unclear locations, despite these deaths logically all taking place within the current IDP camp residences (Supplementary Table S1). Possible explanations include misreporting of the date of arrival to the camp; gradual arrival of parts of the household, with some dying before joining their family in the camp; inclusion of deaths among extended family members who remained outside the camp; household members traveling back to their home villages and dying there; and deaths occurring outside the recall period but being reported within the survey window due to inaccurate date recall and/or ‘telescoping’, a cognitive bias whereby people recall traumatic events as having occurred more recently than they did (52). Altogether, these potential biases may have resulted in over-estimation of baseline period mortality. Additionally, some overreporting of deaths may have occurred during the baseline survey, as households might have anticipated humanitarian assistance in response to reported deaths. This was evidenced by high initial death reports, followed by refusals to participate in verbal autopsy interviews when further details were requested. To mitigate this, community workers were trained to detect inconsistencies through repeated questions, minimizing the risk of misclassification bias (53).

Although verbal autopsy provided valuable insights into mortality trends, methodological challenges remain. Verbal autopsy data and underlying diseases were based on family self-reports, which we could not verify. Given the need to translate interviews into Somali, potential language discrepancies and recall biases may have contributed to misclassification errors (54). Lastly, the lack of unique identifiers for individuals limits the accuracy of person-time calculations, as we relied on household-level IDs and the mid-point of the recall period. Without individual tracking, variations in entry and exit dates could not be precisely accounted for, potentially leading to over- or underestimation of mortality and migration rates. Using unique identifiers would improve the accuracy of these estimates and allow better tracking across survey rounds.

## Conclusion

The ongoing humanitarian crisis in Somalia, exacerbated by El Niño-induced flooding, severe food shortages, and displacement-related health vulnerabilities, continues to strain an already fragile healthcare system. Given these challenges, improving mortality assessments is essential for guiding effective humanitarian responses. However, we acknowledge that a comprehensive, nationwide mortality survey is not immediately feasible due to security constraints, operational barriers, and funding limitations. Instead, alternative methodologies such as community-based surveillance, sentinel site monitoring, remote sensing data, and small-area estimation techniques offer practical and scalable approaches to capturing mortality trends beyond IDP camps. These methods, successfully used in other conflict-affected settings, can provide robust mortality data while minimizing security risks. Our findings highlight critical gaps in healthcare accessibility, disease surveillance, and trauma care, particularly within overcrowded and resource-limited IDP camps.

While Somalia’s mortality patterns align with broader conflict-affected trends, localized factors—including displacement-related malnutrition, weak transport infrastructure, and healthcare fragmentation—exacerbate the crisis. Addressing these issues requires a realistic, context-driven response that considers Somalia’s volatile security landscape and shifting donor commitments. To enhance humanitarian effectiveness, we propose localized health system strengthening through community-based health interventions, mobile clinics, and NGO-led initiatives; humanitarian negotiation and engagement to facilitate limited healthcare access in contested areas through neutral third-party mediation; and adaptation to funding constraints by engaging alternative donors, public-private partnerships, and diaspora-driven health initiatives in light of the US aid freeze. Given Somalia’s complex humanitarian landscape, a flexible and phased approach is essential to maximize impact, mitigate security risks, and sustain critical health interventions. By integrating innovative data collection methodologies with adaptive intervention strategies, humanitarian actors can improve mortality estimation and targeted response efforts, ultimately reducing preventable deaths and enhancing health system resilience in one of the world’s most challenging conflict settings.

## Data Availability

The original contributions presented in the study are included in the article/Supplementary material, further inquiries can be directed to the corresponding author.
