# Correction: Community-based mortality surveillance among internally displaced vulnerable populations in Banadir region, Somalia, 2022–2023

**DOI:** 10.3389/fpubh.2026.1803865

**Published:** 2026-02-16

**Authors:** Frontiers Production Office

**Affiliations:** Frontiers Media SA, Lausanne, Switzerland

**Keywords:** Somalia, humanitarian crisis, internally displaced persons, community-based surveillance, verbal autopsy, mortality rates, malnutrition, infectious diseases

Reference 13 was erroneously written given the accession date September 25, 2025. It should be September 25, 2024.

The original version of this article has been updated.

