# Supplementary material for: Community-based mortality surveillance among internally displaced vulnerable populations in Banadir region, Somalia, 2022–2023
Source: Front Public Health. 2025 Apr 9;13:1582558. doi: 10.3389/fpubh.2025.1582558 (PMC12014603; doi:10.3389/fpubh.2025.1582558)
Supplement: Supplementary file 1 [file Data_Sheet_1.pdf]

Supplementary materials

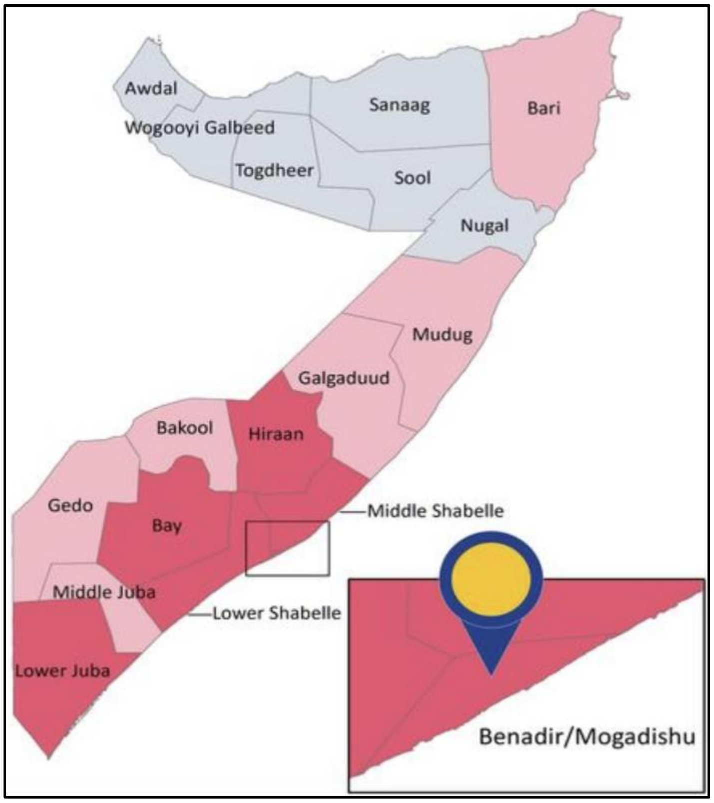

Supplementary Figure 1a: Map showing Somalia Map, Benadir Region and Kahda/Daynile districts

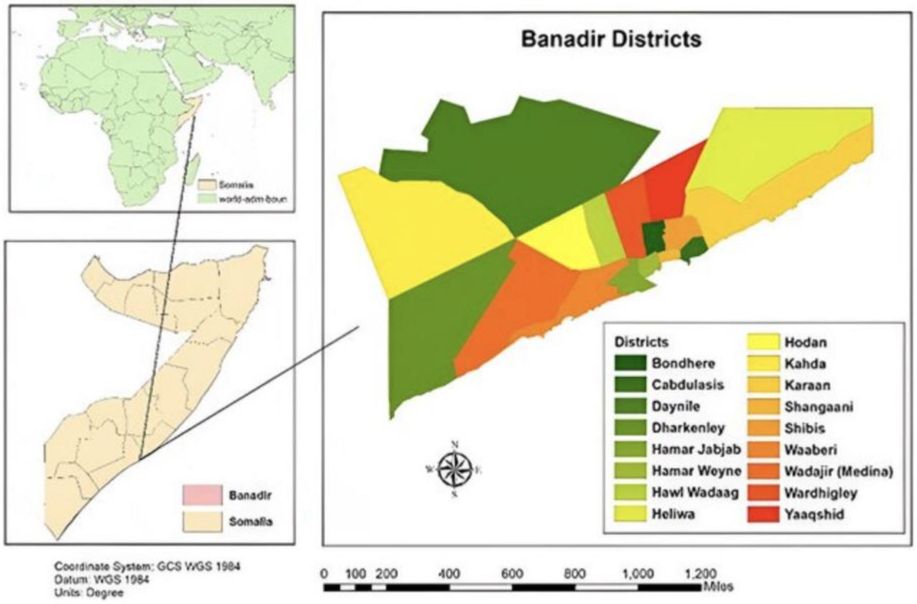

Supplementary Figure 1b: Map showing Benadir Region

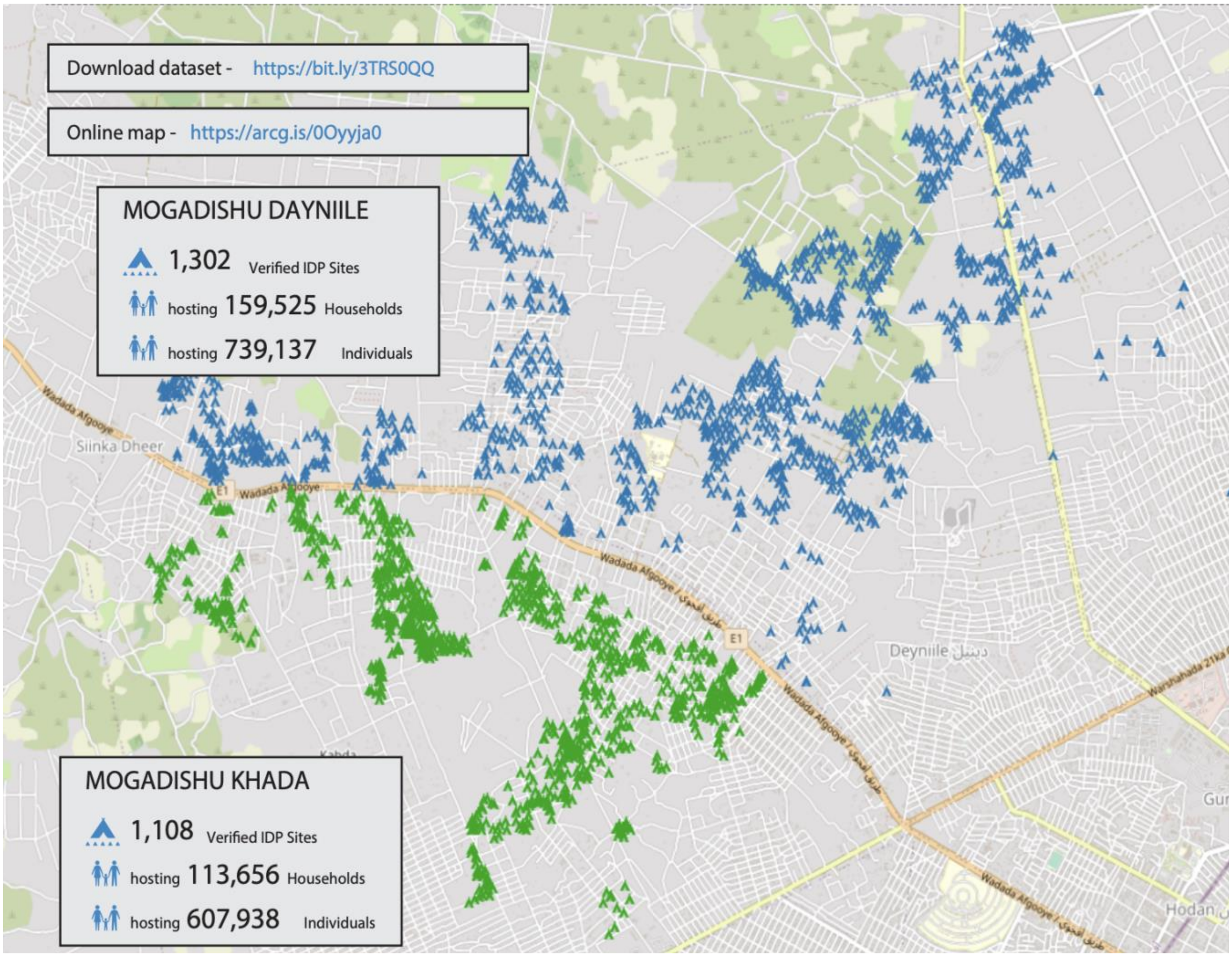

Figure 1c: CCCM Cluster Somalia - Mogadishu Dayniile and Khada Verified IDP Sites - October 2022

Description of the population

The age and sex distribution of individuals by location and outcome across the three prospective surveillance rounds is presented in Figure S1. Mortality ("Died") was concentrated among vulnerable groups, particularly children under five years and adults over 60 years, highlighting the disproportionate impact of mortality on these populations. New entrants into the study area ("Joined") were predominantly younger adults and middle-aged individuals, reflecting migration or displacement trends likely driven by conflict, food insecurity, or economic pressures. Similarly, individuals who left the study area ("Left") were primarily younger adults, indicating that mobility was associated with this demographic. In contrast, the "Remained" category exhibited a broader and more stable age distribution, encompassing individuals across all age groups, which reflects a relatively enduring resident population.

The patterns remained consistent across the three surveillance rounds, with minor variations observed. For instance, the density of the "Left" category narrowed in later rounds, possibly indicating reduced migration over time. The age and sex distributions for males and females were largely similar across all outcomes, with no significant sex-specific differences, although subtle variations in density suggest possible gendered dynamics in mobility or vulnerability. These findings highlight critical demographic patterns in mortality, migration, and population stability, underscoring the need for targeted public health interventions tailored to these age-specific and mobility-related trends.

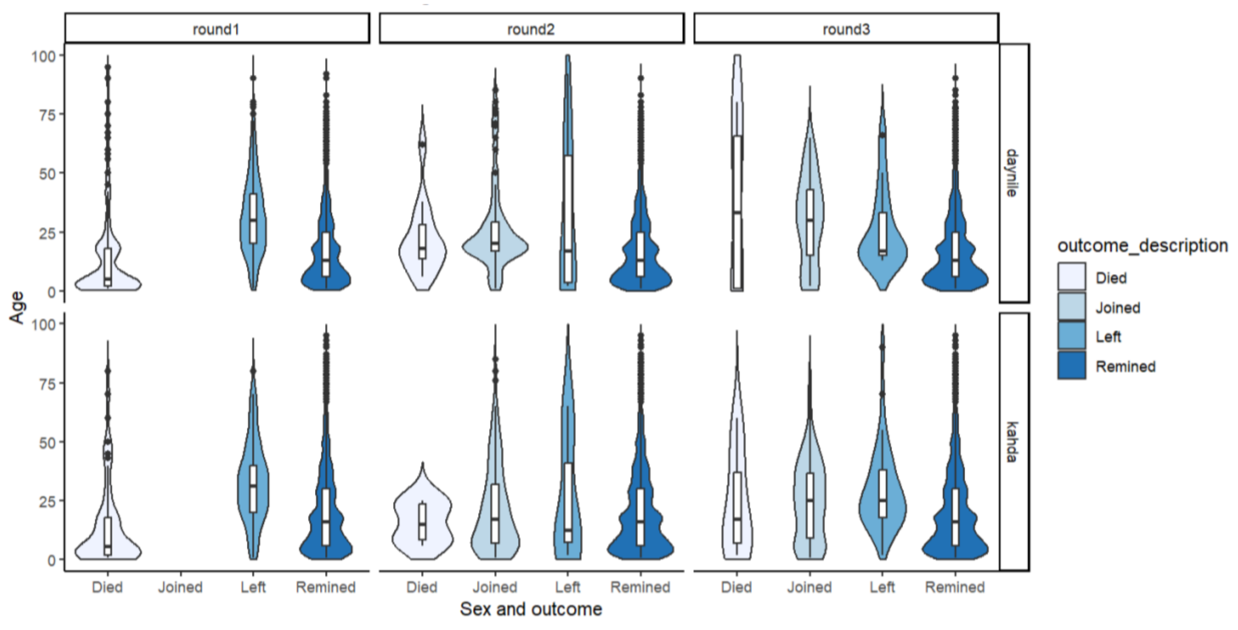

Supplementary Figure S2. Age and sex distribution by location, outcome and round (period).

Supplementary table S1: distribution of 722 recorded deaths across four critical phases of displacement.

**Supplementary table S1**	
Location	Number of deaths (%)
Current location	468 (64.9%)
During migration	124 (17.2%)
Other / Unclear	9 (1.2%)
Previous residence	121 (16.7%)
Total	722

1. Verbal autopsy

Supplementary Figures S3 and S4 provide a detailed breakdown of mortality trends, highlighting the urgent need for age-specific, targeted interventions to reduce preventable deaths. Mortality patterns among neonates (0–28 days) and individuals over five years reveal distinct yet interrelated health challenges.

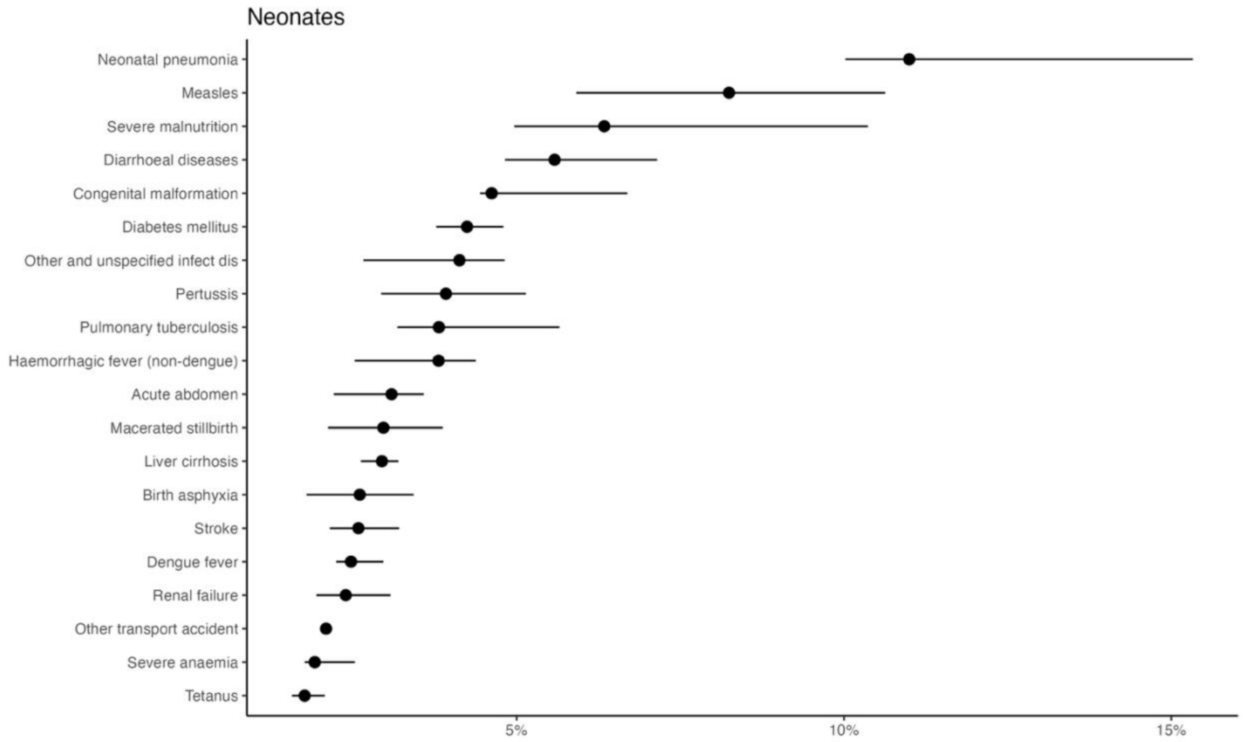

Supplementary Figure S3: Mortality patterns among neonates (0–28 days)

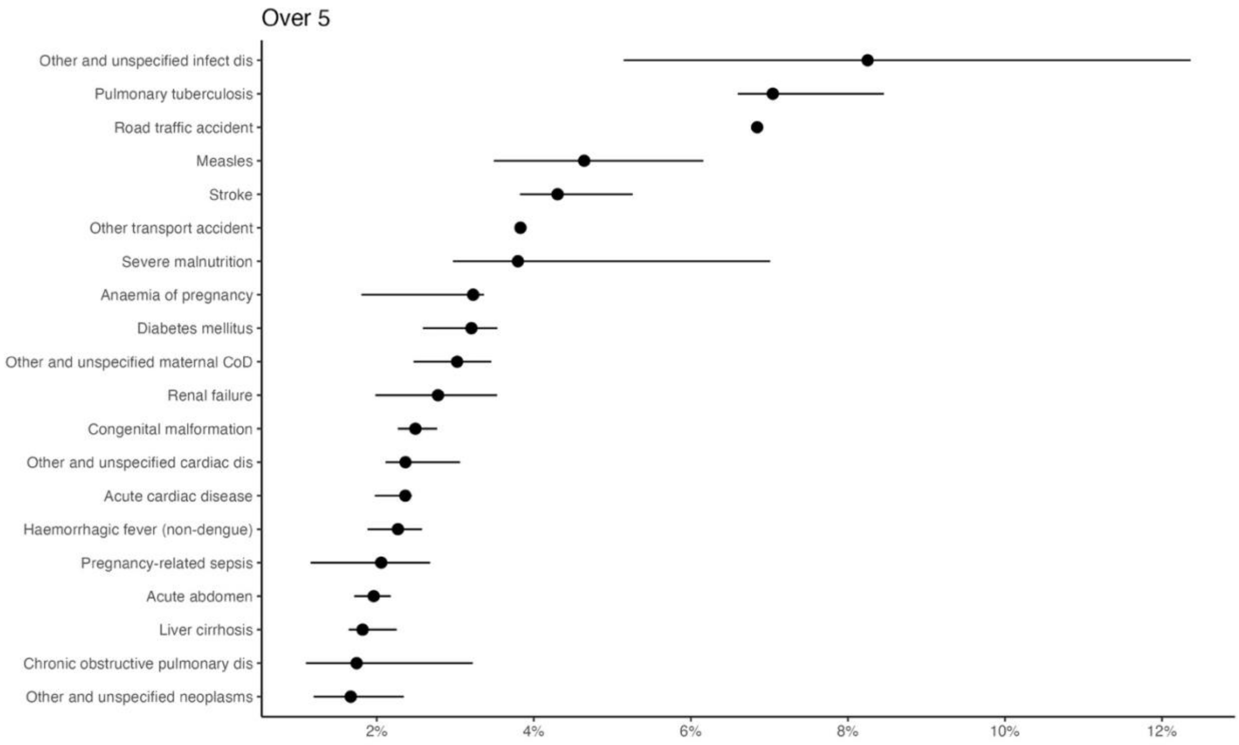

Supplementary Figure S4: Mortality patterns among (>5 years)
